# Circulating prekallikrein levels are correlated with lipid levels in the chinese population: a cross-sectional study

**DOI:** 10.1186/s12944-023-01849-5

**Published:** 2023-06-23

**Authors:** Yuanlu Shu, Xiang Zhao, Changshun Yang, Yan Yan, Yao Zheng, Xijie Wang, Chengfeng Qiu

**Affiliations:** 1 Evidence-based Medicine and Clinical Center, The First People’s Hospital of Huaihua, Huaihua, 418000 P.R. China; 2 Department of General Practice, The First People’s Hospital of Huaihua, Huaihua, 418000 P.R. China; 3 Department of Laboratory Medicine, The First People’s Hospital of Huaihua, Huaihua, 418000 P.R. China; 4 School of Public Health and Laboratory Medicine, Hunan University of Medicine, Huaihua, 418000 P.R. China; 5 College of Biology and Environmental Sciences, Jishou University, Jishou, 416000 P.R. China; 6 Department of Clinical Pharmacy, The First People’s Hospital of Huaihua, Huaihua, 418000 P.R. China; 7 School of Basic Medical Sciences, University of South China, Hengyang, 421000 P.R. China

**Keywords:** Prekallikrein, Hypercholesterolemia, Hypertriglyceridemia, Lipids, Cardiovascular diseases

## Abstract

**Background:**

Recent evidence has revealed that circulating coagulation factor prekallikrein (PK), an important part of the kallikrein-kinin system, regulates cholesterol metabolism, but the association between serum PK and lipid levels is unclear.

**Methods:**

This cross-sectional study included 256 subjects (aged from 1 month to 90 years) who underwent physical examinations at the First People’s Hospital of Huaihua, China. After overnight fasting, serum was collected for PK and lipid testing. Spearman correlation analysis and multivariable logistic regression analysis were used to analyze the association of PK level with lipid levels and the likelihood risk of hyperlipidemia. The possible threshold value of PK was calculated according to the receiver operating characteristic (ROC) curve.

**Results:**

The median serum PK level was 280.9 µg/mL (IQR 168.0, 377.0), and this level changed with age but not sex. The serum PK level was positively correlated with the serum total cholesterol (TC), low-density lipoprotein cholesterol (LDL-C), and triglyceride (TG) levels. A nonlinear relationship was observed between serum PK and high-density lipoprotein cholesterol (HDL-C) levels. The serum PK level was positively correlated with HDL-C when its level was lower than 240 µg/mL and negatively correlated with HDL-C when its level was higher than 240 µg/mL. The regression analysis demonstrated that an elevated serum PK level was significantly associated with the likelihood risk of hypercholesterolemia and hypertriglyceridemia. The ROC curve showed that the possible threshold values of serum PK for hypercholesterolemia and hypertriglyceridemia occurrences were 344.9 µg/mL and 305.7 µg/mL, respectively.

**Conclusions:**

Elevated serum PK levels were significantly associated with the likelihood of hypercholesterolemia and hypertriglyceridemia, and the possible threshold values of PK levels were 344.9 µg/mL and 305.70 µg/mL, respectively, suggesting that higher PK levels may be a risk factor for cardiovascular diseases.

**Supplementary Information:**

The online version contains supplementary material available at 10.1186/s12944-023-01849-5.

## Introduction

Prekallikrein (PK), a single-stranded glycoprotein encoded by the KLKB1 gene, is composed of 800 amino acids and has a molecular weight of 88 kDa [1]. PK is a serine protease that is mainly synthesized in the liver [2, 3]. PK plays a crucial role in the kallikrein-kinin system (KKS) and can initiate the intrinsic coagulation pathway. PK activates coagulation factor XII (FXII) to become active FXII (FXIIa), and in turn, FXIIa converts PK into its active form plasma kallikrein (PKa) [4]. PKa is composed of a heavy chain (371 amino acids) and a light chain (248 amino acids), promoting the production of FXIIa and thereby expanding the coagulation cascade [1]. Furthermore, PK regulates various physiological pathways, such as fibrinolysis, inflammation, and complements in the immune response. Therefore, PK is considered a potential therapeutic target for some diseases, including diabetes microvascular complications, hereditary angioedema, and cardiovascular and cerebrovascular diseases.

Some evidence has shown that PK is associated with cardiovascular diseases (CVDs) [5–7]. Hyperlipidemia is a major risk factor for CVD. Recently, a study revealed that PK regulates cholesterol metabolism by binding to low-density lipoprotein receptors (LDLRs) and inducing LDLR lysosomal degradation [8]. The serum level of PK is positively correlated with low-density lipoprotein cholesterol (LDL-C), total cholesterol (TC), and triglyceride (TG) but not with high-density lipoprotein cholesterol (HDL-C) in young adult Han Chinese (aged 17–25 years). A previous study also demonstrated similar correlations in children aged 9–11 years [9]. This means that an increase in serum PK levels leads to increased lipid levels, thus leading to an increased occurrence of hyperlipidemia. However, those two studies have only evaluated the correlations in a certain age group. Therefore, a total of 256 subjects aged 1 month to 90 years were included to identify the associations of serum PK with lipid levels and the occurrence of hyperlipemia.

## Methods

### Participants

This cross-sectional study was approved by the First People’s Hospital of Huaihua Ethics Commission. From March 2022 through June 2022, a total of 256 subjects (age range 1 month to 90 years) received physical examinations at the Medical Examination Centre and Child Health Outpatient. Exclusion criteria: (1) subjects were diagnosed with any acute or chronic disease or severely restricted renal and liver function; (2) pregnant females; (3) subjects were taking lipid-lowering therapy (use lipid-lowering drugs within at least one week before recruitment). Obtain written consent from the parents or guardians of adults or minors/children. Key demographic and clinical characteristics were recorded at the time of enrollment.

### Outcomes

According to the “Guidelines for Prevention and Treatment of Dyslipidemia in Chinese Adults” [10], hypercholesterolemia was defined as a fasting serum level of TC higher than 5.2 mmol/L (240 mg/dL), and hypertriglyceridemia was defined as a fasting serum level of TG higher than 1.7 mmol/L (150 mg/dL).

### Blood sample collection and laboratory measurements

Peripheral venous blood samples were collected at 7 am after overnight fasting for routine clinical detection and PK testing. Collected blood samples were centrifuged at 1000 × g for 15 min, and then the serum was separated and stored at -80 °C until further analysis. Blood lipid profiles were measured using an Olympus AU5400 (Beckman-Coulter) Automated Clinical Chemistry Analyses. Serum PK levels were quantified using the Human PK Enzyme-Linked Immunosorbent Assay (ELISA) Kit (Abcam, ab202405). The Human PK ELISA Kit is an ELISA kit commonly used for in vitro quantitative measurement of PK concentrations in human serum. This PK kit has shown high sensitivity and specificity [11, 12]. All the testing steps were based on the kit specifications.

### Statistical analysis

The Shapiro‒Wilk test was used to test the normality of continuous variables. Normally distributed variables are presented as the mean ± standard deviation (SD), and nonnormally distributed variables are presented as the median (interquartile range, IQR). Categorical variables are presented as numbers and percentages. Continuous outcomes were compared using the Wilcoxon rank-sum test or the Kruskal‒Wallis test. The correlation between PK and lipid parameters was analyzed using the Spearman correlation. Using the PK level as either a continuous or a categorical variable, a multivariable logistic regression analysis was performed to explore its association with hyperlipidemia. The results are presented as odds ratios (ORs) and 95% confidence intervals (CIs). As a continuous variable, PK was standardized (mean = 0, SD = 1), and then the impact of a 1-SD increase was assessed. As a categorical variable, PK was divided into tertiles. The subjects in the bottom tertile were defined into the reference group, and those in the other tertiles were defined into the risk group, and differences in linear trends were tested. Receiver operating characteristic (ROC) curve analysis was used to determine a threshold of PK to identify hypercholesterolemia and hypertriglyceridemia. A *P* value < 0.05 indicated statistical significance.

## Results

### Characteristics of the study population

A total of 256 subjects, including 136 males and 120 females, were recruited in the study (median age 36 years [IQR, 17–58], range 1 month to 90 years) (see Additional file: Figure S1). Among them, 208 subjects (age > 14 years) were recruited from the Medical Examination Centre, and 48 subjects (age ≤14 years) were recruited from the Child Health Outpatient. In those aged > 14 years, 70 (33.65%) and 72 (34.62%) subjects were diagnosed with hypercholesterolemia and hypertriglyceridemia, respectively. The median serum PK was 280.9 µg/mL (IQR 168.0, 377.0) (Table 1).

**Table 1:** Baseline characteristics of study subjects

Characteristics	All (*N* = 256)
Age, year	36 (17,58)
Sex, n (%)	
Female	120 (46.88)
Male	136 (53.12)
BMI, kg/m^2^	22.86 ± 2.22^a^
Hypercholesterolemia, n (%)	70 (33.65)^a^
Hypertriglyceridemia, n (%)	72 (34.62)^a^
TC, mmol/L	4.49 (3.68, 5.31)
LDL-C, mmol/L	2.39 (1.85, 3.06)
HDL-C, mmol/L	1.21 (1.01, 1.39)
TG, mmol/L	1.31 (0.91, 1.91)
PK, µg/ml	280.9 (168.0, 377.0)

^a^208 subjects aged 15–90 years

BMI, body mass index; TC, total cholesterol; LDL-C, low-density lipoprotein cholesterol; HDL-C, high-density lipoprotein cholesterol; TG, triglyceride; PK, prekallikrein

### Distribution of serum PK levels

**Fig. 1:**
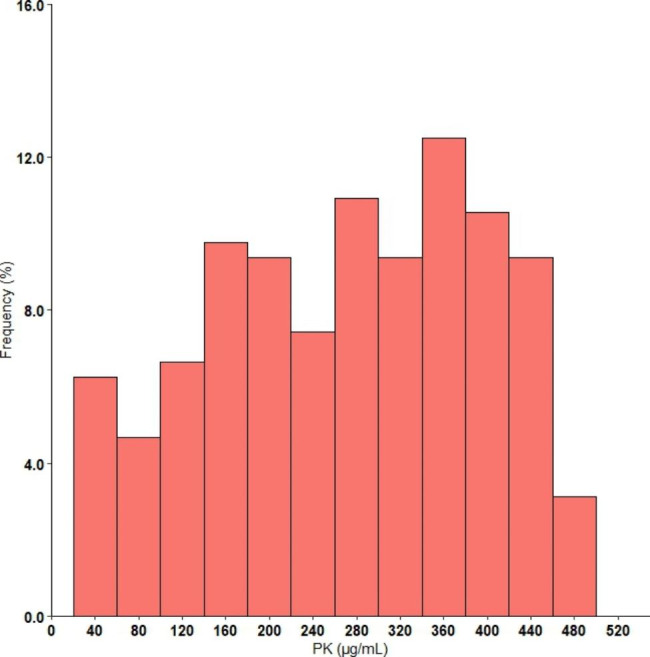
Distribution of fasting serum PK values, n = 256

**Fig. 2:**
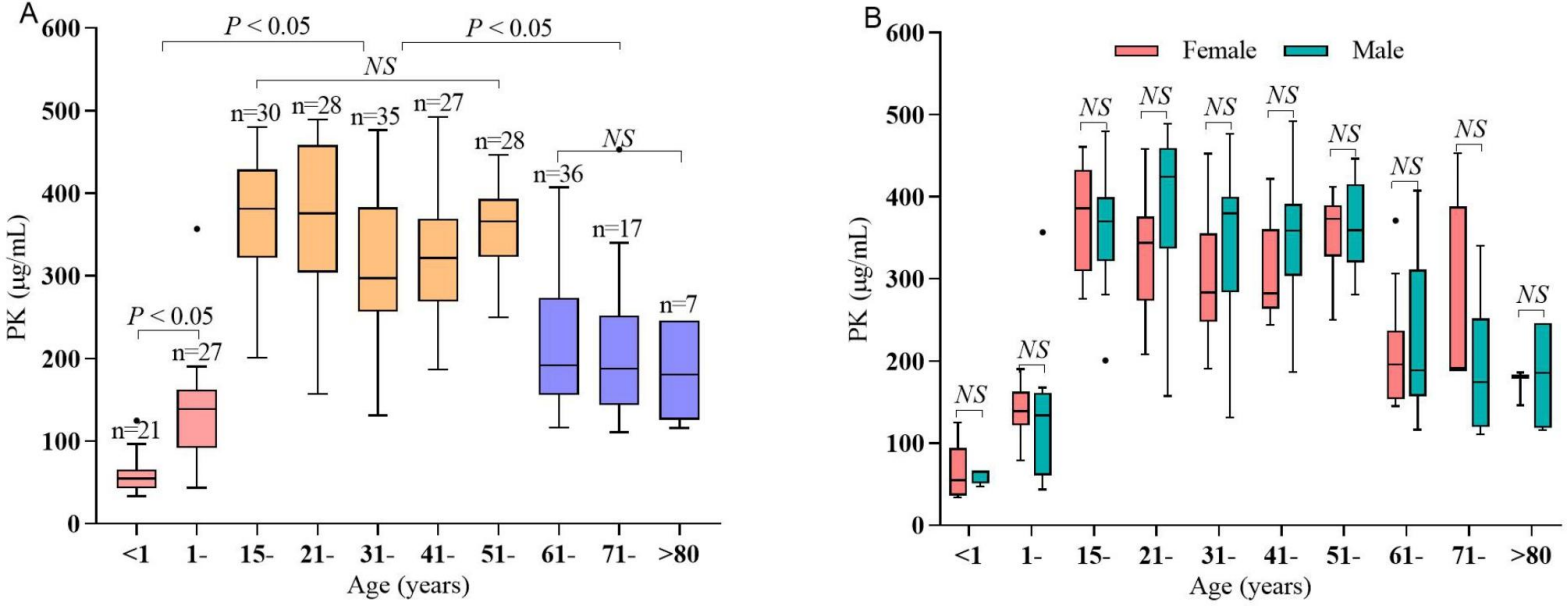
Distribution of serum PK in all participants grouped by age **(A)** and sex **(B)** differences

Fasting serum PK levels showed a right-skewed distribution with a range from 33.3 to 491.9 µg/mL (Fig. 1). Interestingly, the results showed an obvious association between age and serum PK (Fig. 2a). The serum PK level increased from 54.6 to 138.6 µg/mL in the subjects aged 1 month to 14 years, but it varied very little in the subjects aged 15–60 years. Subsequently, a significant decreasing trend in serum PK was observed in the older subjects aged 61–90 years. The median serum PK levels in the three groups were 85.0 µg/mL (IQR 54.0, 139.4), 358.5 µg/mL (IQR 287.5, 398.9), and 188.0 µg/mL (IQR 153.8, 245.9), respectively, *P* < 0.001. Furthermore, similar age-related associations appeared in serum TC, LDL-C, and TG levels but not in HDL-C levels (see Additional file: Figure S2). However, the sex-caused difference in PK was not found in each age group (Fig. 2b).

### Serum PK levels were correlated with lipid levels

**Fig. 3:**
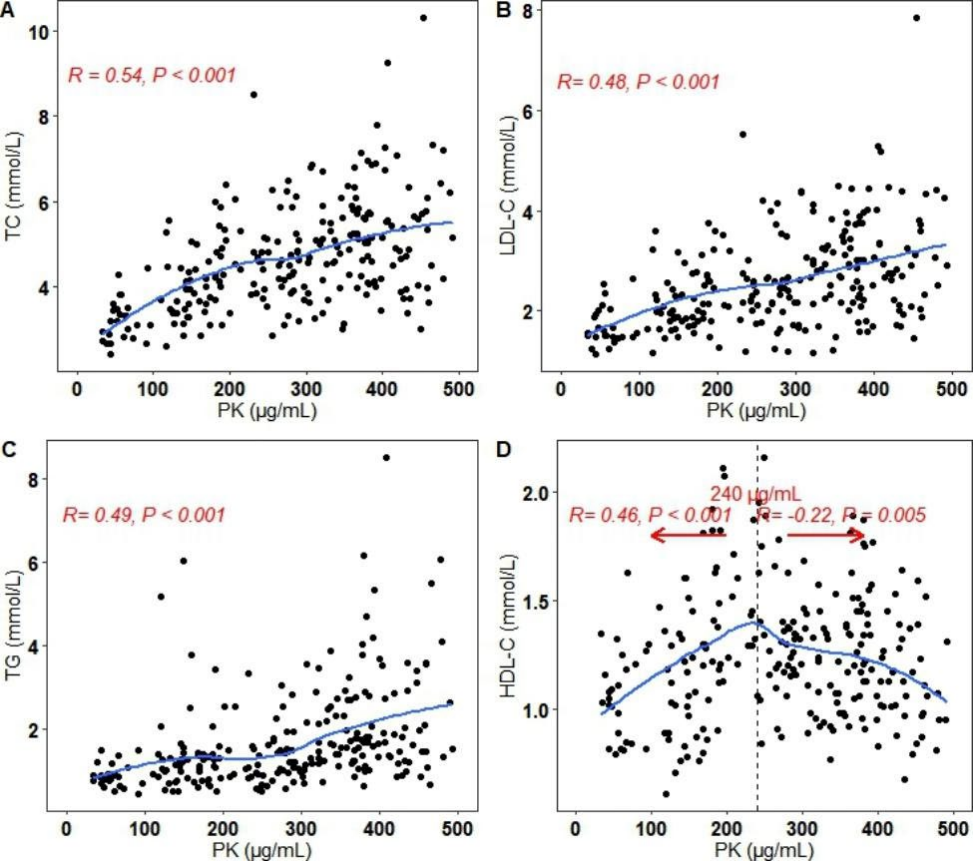
Correlations between serum PK and TC **(A)**, LDL-C **(B)**, TG **(C)**, and HDL-C levels **(D)**. The correlation was calculated using the Spearman correlation coefficient (R). A linear relationship was observed between PK and TC, LDL-C and TG, and a nonlinear relationship was observed between PK and HDL-C.

Serum PK levels were positively correlated with TC (R = 0.54, *P* < 0.001), LDL-C (R = 0.48, *P* < 0.001), and TG (R = 0.49, *P* < 0.001) levels (Fig. 3). Subgroup analysis showed the same correlations in the 15–60 years and 61–90 years groups (see Additional file: Figure S3, 4). In the 1 month-14 years group, the positive correlations between serum PK and TC and LDL-C remained obvious, but that between serum PK and TG was not observed (see Additional file: Figure S5).

**Fig. 4:**
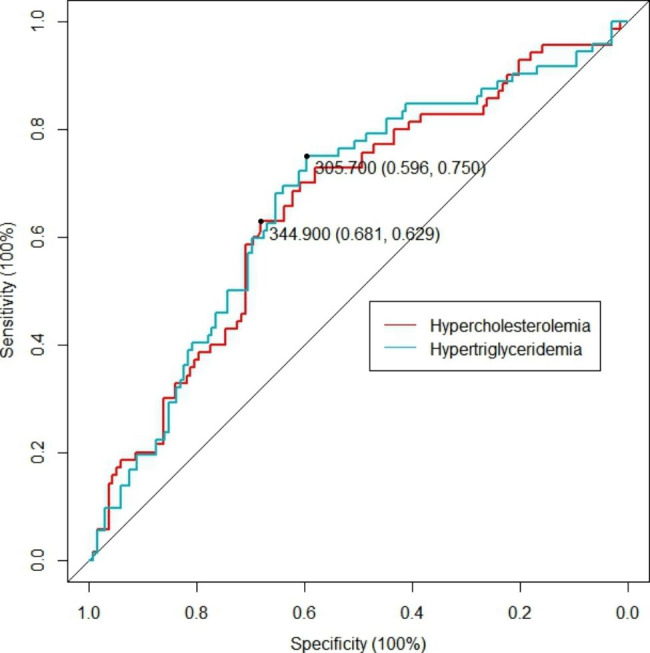
ROC analysis of the relationship between PK and hyperlipidemia. The optimal cutoff values of hypercholesterolemia and hypertriglyceridemia were 344.9 µg/mL and 305.7 µg/mL, respectively

An inverse U-shaped association between serum PK and HDL-C was found in all subjects (Fig. 3). Serum PK was positively correlated with HDL-C when it was lower than 240 µg/mL and negatively correlated with serum HDL-C when it was higher than 240 µg/mL. In the subgroup analysis, a similar negative correlation was found in the subjects aged 15–60 years but not in the other subjects (see Additional file: Figure S3-5).

### Elevated serum PK was associated with hyperlipidemia

Children aged ≤14 years were not considered in the logistic regression analysis due to the lack of unified diagnostic criteria for pediatric hypercholesterolemia and hypertriglyceridemia. The logistic regression analysis showed that serum PK, if taken as a continuous variable, was significantly associated with hypercholesterolemia (OR 1.78 [95% CI: 1.31, 2.48]) and hypertriglyceridemia (OR 1.83 [95% CI: 1.34, 2.54]) (Table 2). After adjusting for confounders, these associations remained significant.

**Table 2:** Association between PK level and hyperlipidemia

	Case/N	Hypercholesterolemia		Case/N	Hypertriglyceridemia	
		OR (95% CI)	OR_adj_ (95% CI)		OR (95% CI)	OR_adj_ (95% CI)
Pre-SD increase	70/208	1.78(1.31, 2.48)	3.14(2.03, 5.09)	72/208	1.83(1.34, 2.54)	2.41(1.60, 3.73)
1^st^ T	13/69	1	1	12/69	1	1
2^nd^ T	26/70	1.38(0.62, 3.16)	5.29(1.69, 19.35)	26/70	1.74(0.78, 4.04)	4.03(1.36, 13.28)
3^rd^ T	31/69	2.99(1.39, 6.77)	13.13(3.94, 51.98)	34/69	3.52(1.62, 8.15)	8.03(2.60, 27.88)
*P* trend		0.004	< 0.001		0.001	< 0.001

Adjusting for age, sex, and BMI.

When the serum PK level was analyzed as a categorical variable, the middle tertile (OR 5.29 [95% CI: 1.69, 19.35]) and top tertile (OR 13.13 [95% CI: 3.94, 51.98]) were associated with the occurrence of hypercholesterolemia compared to the bottom tertile after adjusting for age, sex, and BMI. Similar associations were also observed in hypertriglyceridemia (OR 4.03 [95% CI: 1.36, 13.28] and OR 8.03 [95% CI: 2.60, 27.88], respectively). Specifically, the subjects with a higher PK level, rather than a lower level, were more likely to have hypercholesterolemia and hypertriglyceridemia (*P* trend < 0.001).

Due to the serum PK level being related to age, subgroup analysis was performed (Table 3). A significant interaction was revealed in the subgroups of age (*P* < 0.05). Serum PK levels were associated with both hypercholesterolemia and hypertriglyceridemia, regardless of age.

**Table 3:** Subgroup analysis of age

Subgroup	Hypertriglyceridemia		Hypertriglyceridemia	
	OR (95% CI)	*P* for interaction	OR (95% CI)	*P* for interaction
15–60 years	2.85(1.66, 4.91)	0.002	3.84(1.66, 8.87)	< 0.001
61–90 years	2.37(1.43, 3.92)		2.94(1.40, 6.17)	

Figure 4 shows the ROC curves of serum PK for discriminating hypercholesterolemia and hypertriglyceridemia. Based on the Youden index, a serum PK of 344.9 µg/mL was identified as an optimal cutoff for hypercholesterolemia (sensitivity 75.0% and specificity 59.6%); meanwhile, a serum PK of 305.7 µg/mL was identified as an optimal cutoff for hypertriglyceridemia (sensitivity 62.9% and specificity 68.1%).

## Discussion

A recent study reported that the serum coagulation factor PK regulates cholesterol metabolism [8]. This study fully estimated the relationship between serum PK and lipid levels. Three major findings were achieved. First, the serum PK level changed largely with age but not with sex. Second, the serum PK level was closely correlated with the serum lipid levels. Third, an elevated serum PK level was associated with a higher likelihood of hyperlipidemia, and the possible threshold values of PK levels for hypercholesterolemia and hypertriglyceridemia occurrences were 344.9 µg/mL and 305.7 µg/mL, respectively.

To fully explore the distribution of serum PK levels in Chinese individuals, the serum PK level was detected in 256 subjects aged 1 month to 90 years, with findings ranging from 33.3 to 491.9 µg/mL. Importantly, the current results showed that the serum PK level increased with age in subjects aged < 14 years, remained almost steady in subjects aged 15–60 years old, and then decreased sharply in elderly individuals aged 61–90 years. The serum PK level is not associated with age in patients with type 1 diabetes [6]. This controversy may come from different study populations. The current study included subjects without any acute or chronic diseases; thus, the results can truly reveal a physiological age-related serum PK distribution. PK is mainly synthesized in the liver; thus, it is speculated that this obvious age-related distribution may result from changes in liver function. Interestingly, similar changes in serum TC, LDL-C, and TG, but not HDL-C levels, with age were also found in this study, indicating the close relationship between serum PK and lipid levels.

The role of serum PK in mediating the intrinsic pathway of coagulation has been well described [13–15]. Recent experimental findings in HEK293T cells and hamsters demonstrated that PK binds hepatic LDLR and induces its lysosomal degradation and an increase in cholesterol levels and further found a positive correlation between PK and cholesterol levels in healthy subjects [8]. This study included participants from all age groups and found positive correlations between serum PK and TC and LDL-C levels. In line with previous studies, these positive correlations are robust. However, this study found a nonlinear correlation between serum PK and HDL-C levels, which was positive when the serum PK level was lower and negative when it was higher than 240 µg/mL. With serum PK > 240 µg/mL in almost 90% of subjects, the group aged 15–60 years presented higher serum PK levels than both the younger and older groups. The subgroup analysis stratified by age demonstrated that serum PK levels were negatively correlated with HDL-C levels in subjects aged 15–60 years, and no obvious association was found in subjects aged < 15 years and 61–90 years. However, the underlying mechanism for this nonlinear correlation is unclear. Nonetheless, these results indicate that age is an important factor that influences serum PK levels under a physiological state.

Studies have demonstrated that serum PK induces LDLR degradation and thus decreases serum cholesterol clearance, suggesting that serum PK is a potential cholesterol-lowering target [8]. In this study, multivariable analysis showed that an elevated serum PK level was associated with a higher likelihood of hypercholesterolemia and hypertriglyceridemia in subjects aged > 14 years, further providing evidence to support that serum PK may be a potential lipid-lowering target. The stratified analysis by age demonstrated similar ORs in the 15–60 years and 61–90 years groups (*P* for interaction was statistically significant).

Epidemiologic and genetic studies have revealed that a high serum PK level may increase CVD risk [5, 6, 16, 17]. However, as an important part of the KKS, PK regulates various physiological pathways, such as coagulation, fibrinolysis, inflammation, and the complement system [18–20]. This study further found that the possible threshold values of serum PK level for predicting hypercholesterolemia and hypertriglyceridemia occurrences were 344.9 µg/mL and 305.7 µg/mL, respectively, which are higher than the median PK level (280.9 µg/mL) in the current study population, suggesting that a higher PK level may be associated with cardiovascular diseases related to lipid dysmetabolism.

### Study strengths and limitations

The current study comprehensively assesses the relationship between PK and lipid levels in individuals aged 1 month to 90 years. The findings highlighted that PK may be associated with cardiovascular diseases related to lipid dysmetabolism. However, some limitations exist in the current study. First, although it included almost all age groups, the age distribution was uneven; the subjects in certain age groups were fewer than those in other groups; for example, there were only 7 subjects in the 81–90 years age group. Second, the causal associations between serum PK and dyslipidemia cannot be fully elucidated. In particular, regarding the role of circulating PK levels in CVD risk assessment, another rigorous prospective study is needed to identify the causes. Third, other components of the KKS were not measured, such as FXII, high molecular weight kininogen (HK), and bradykinin (BK). However, no evidence indicates that the other KKS compounds are related to lipid metabolism.

## Conclusions

This study revealed that the serum PK level changed with age and was closely correlated with lipid levels. An elevated serum PK level is significantly associated with the likelihood of hypercholesterolemia and hypertriglyceridemia, and the possible threshold values of the PK level are 344.9 µg/mL and 305.7 µg/mL, respectively, suggesting that a higher PK level may be associated with cardiovascular diseases related to lipid dysmetabolism.

## Electronic supplementary material

Below is the link to the electronic supplementary material.

**Additional file 1: Figure S1** Distribution of age.

**Additional file 2: Figure S2** Distribution of TC (A), LDL-C (B), TG (C), and HDL-C (D) in different age groups. The Kruskal?Wallis test demonstrated significant differences in TC (P < 0.001), LDL-C (P < 0.001), and TG (P < 0.001) and no significant differences in HDL-C (P = 0.104).

**Additional file 3: Figure S3** Scatter plots depict the association between PK and TC (A), LDL-C (B), TG (C), and HDL-C (D) across ages 15 to 60 years.

**Additional file 4: Figure S4** Scatter plots depict the association between PK and TC (A), LDL-C (B), TG (C), and HDL-C (D) across ages 61 to 90 years.

**Additional file 5: Figure S5** Scatter plots depict the association between PK and TC (A), LDL-C (B), TG (C), and HDL-C (D) across ages 1 month to 14 years.

## Data Availability

Datasets used during the current study are available on reasonable request to the corresponding author.

## Abbreviations

BK: Bradykinin
BMI: Body mass index
CI: Confidence intervals
CVD: Cardiovascular diseases
ELISA: Enzyme-linked immunosorbent assay
FXII: Coagulation factor XII
FXIIa: Activates FXII
HDL-C: High-density lipoprotein cholesterol
HK: High molecular weight kininogen
IQR: Interquartile range
KKS: Kallikrein-kinin system
LDL-C: Low-density lipoprotein cholesterol
LDLR: Low-density lipoprotein receptor
OR: Odds ratios
PK: Prekallikrein
PKa: Plasma kallikrein
ROC: Receiver operating characteristic
SD: Standard deviation
TC: Total cholesterol
TG: Triglyceride
