# Supplementary figures and images for: Circulating prekallikrein levels are correlated with lipid levels in the chinese population: a cross-sectional study
Source: Lipids Health Dis. 2023 Jun 23;22:79. doi: 10.1186/s12944-023-01849-5 (PMC10290373; doi:10.1186/s12944-023-01849-5)

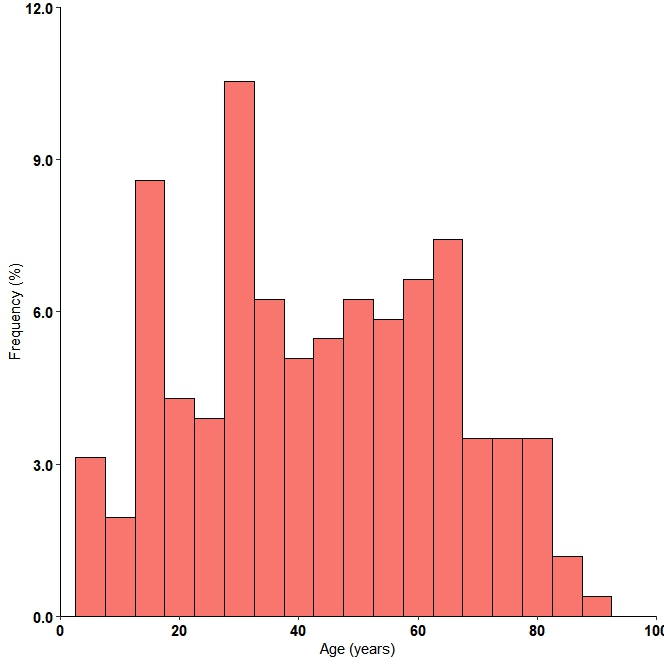

Supplement: Supplementary file 1 — Additional file 1: Figure S1 Distribution of age. [file 12944_2023_1849_MOESM1_ESM.jpeg]

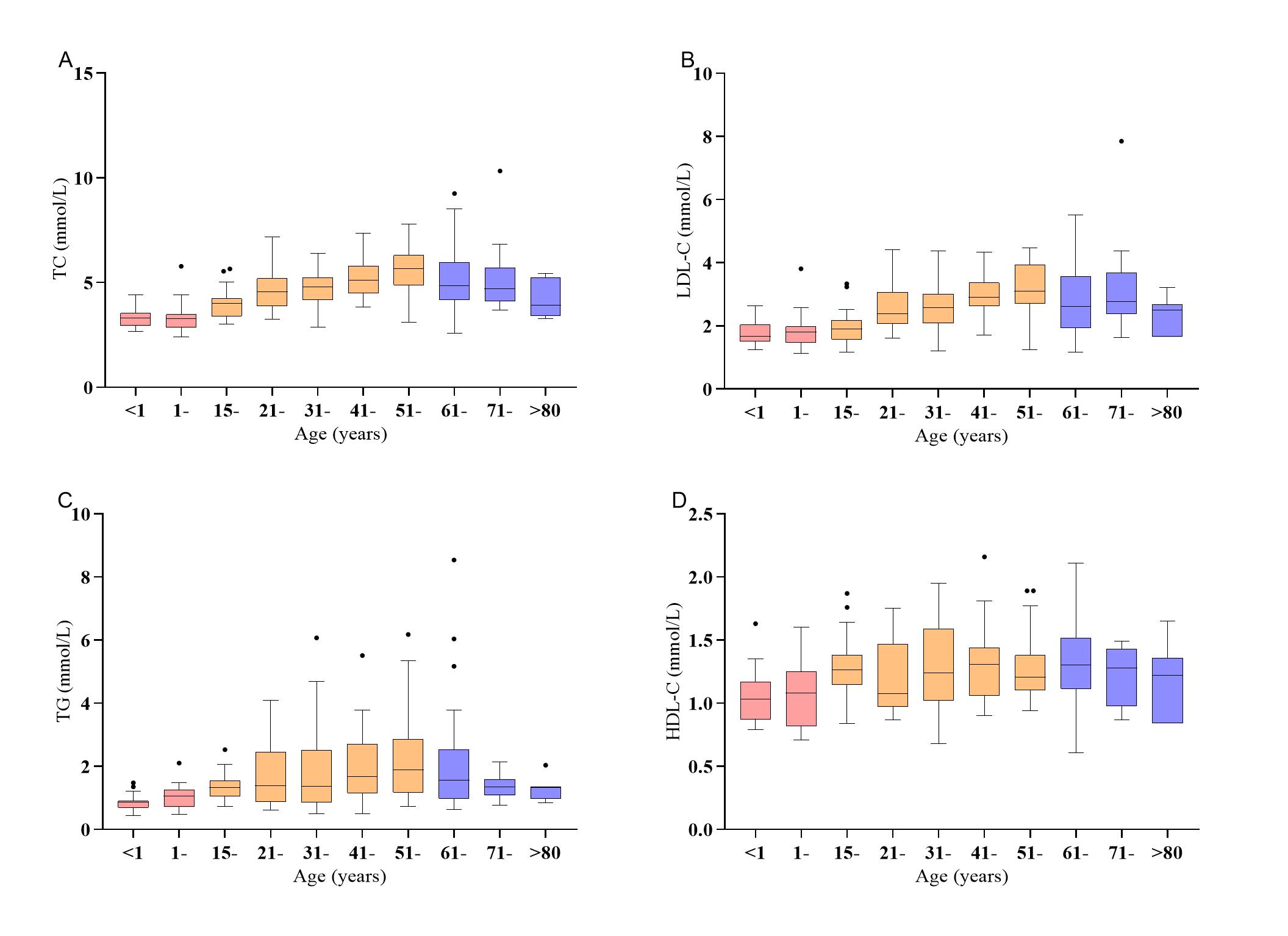

Supplement: Supplementary file 2 — Additional file 2: Figure S2 Distribution of TC (A), LDL-C (B), TG (C), and HDL-C (D) in different age groups. The Kruskal?Wallis test demonstrated significant differences in TC (P < 0.001), LDL-C (P < 0.001), and TG (P < 0.001) and no significant differences in HDL-C (P = 0.104). [file 12944_2023_1849_MOESM2_ESM.jpg]

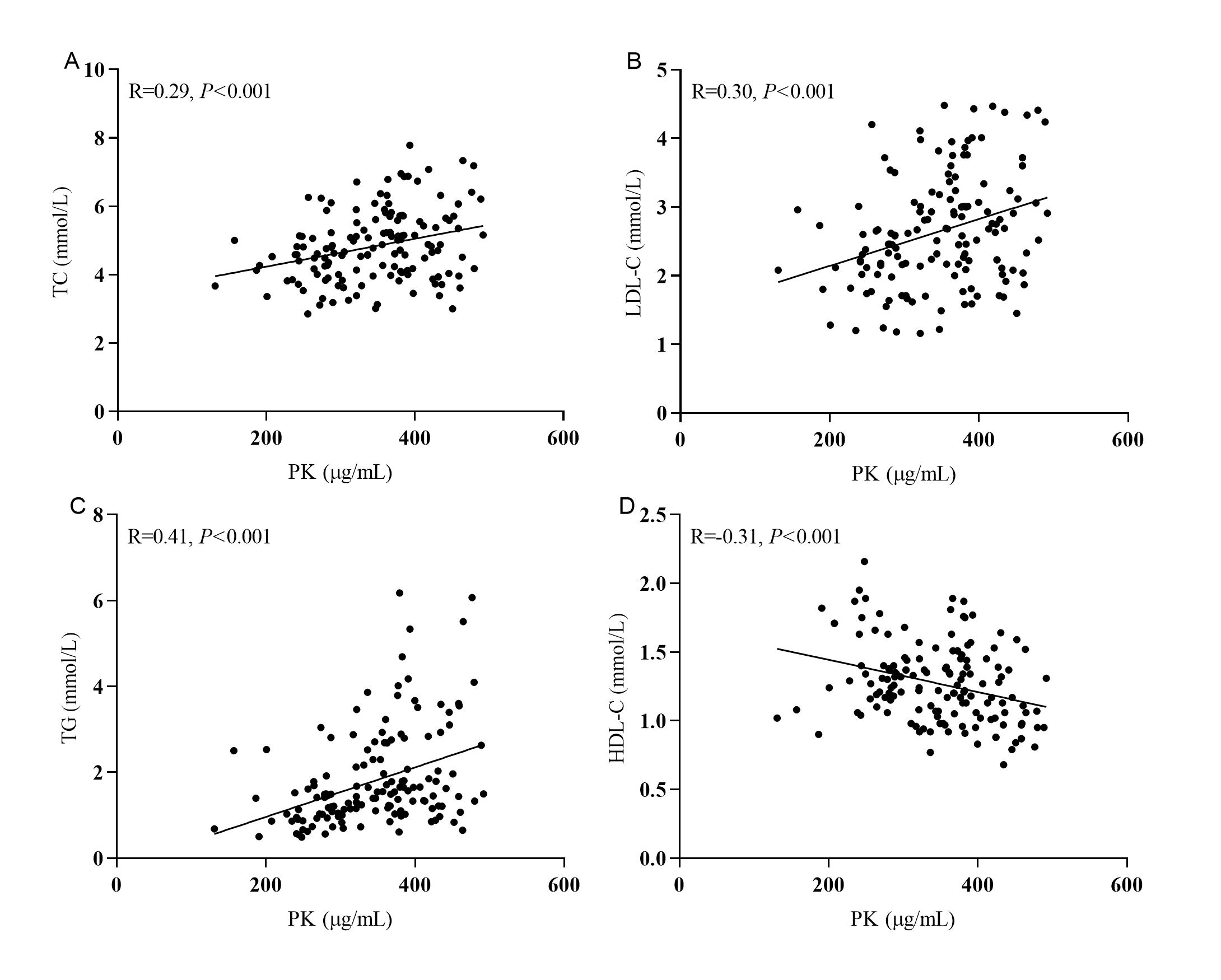

Supplement: Supplementary file 3 — Additional file 3: Figure S3 Scatter plots depict the association between PK and TC (A), LDL-C (B), TG (C), and HDL-C (D) across ages 15 to 60 years. [file 12944_2023_1849_MOESM3_ESM.jpg]

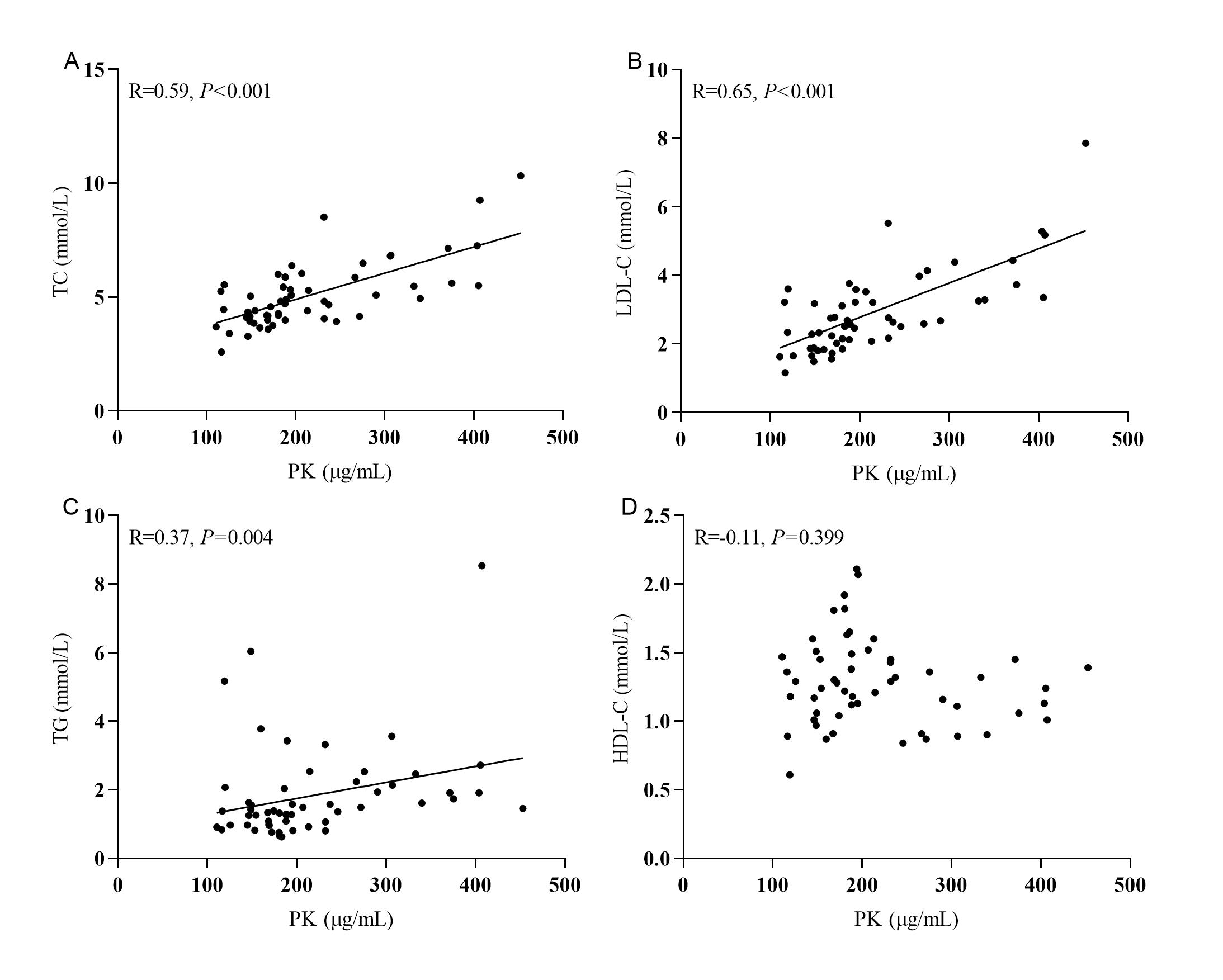

Supplement: Supplementary file 4 — Additional file 4: Figure S4 Scatter plots depict the association between PK and TC (A), LDL-C (B), TG (C), and HDL-C (D) across ages 61 to 90 years. [file 12944_2023_1849_MOESM4_ESM.jpg]

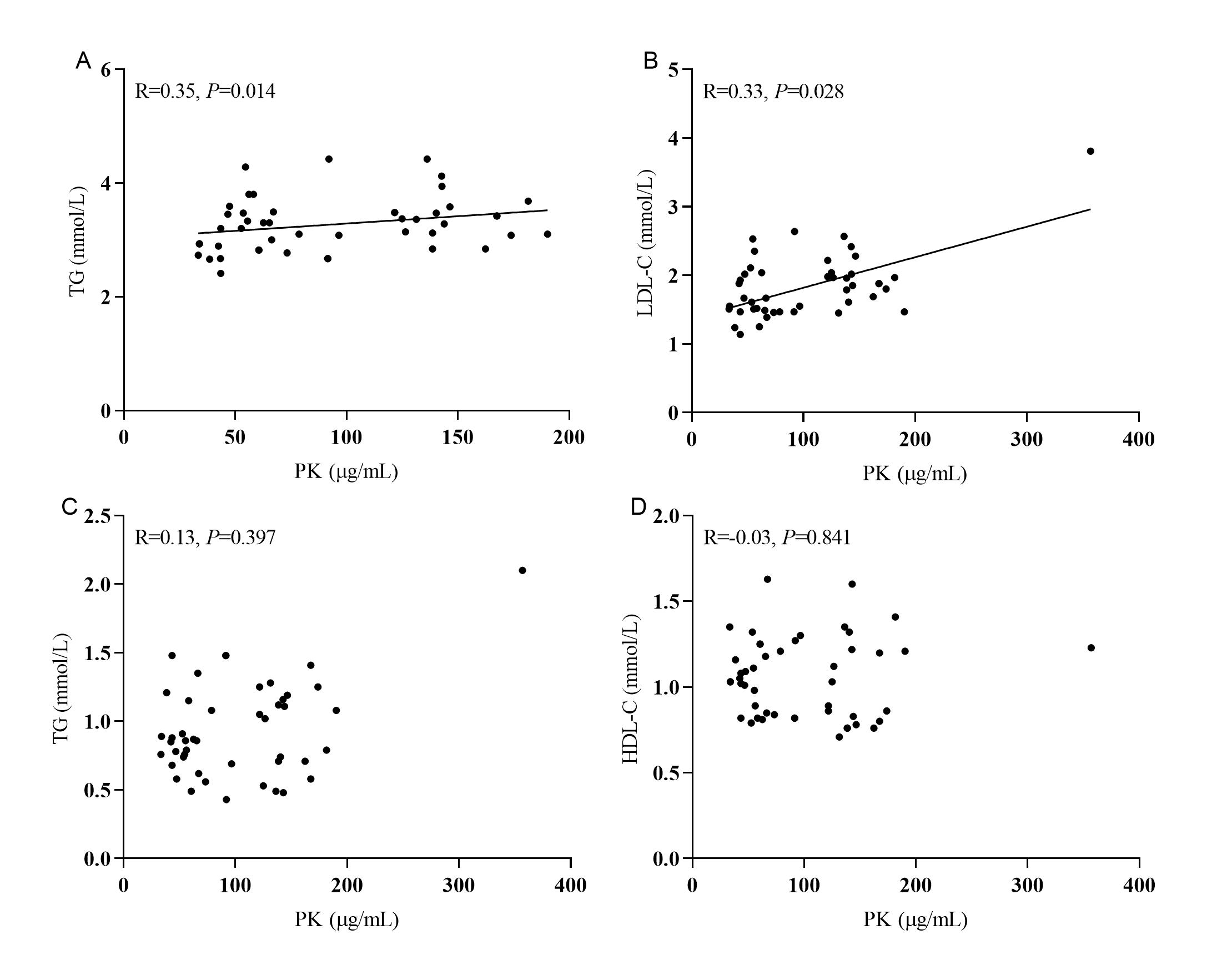

Supplement: Supplementary file 5 — Additional file 5: Figure S5 Scatter plots depict the association between PK and TC (A), LDL-C (B), TG (C), and HDL-C (D) across ages 1 month to 14 years. [file 12944_2023_1849_MOESM5_ESM.jpg]
